# By What Process Would Registration Contribute to the Prosperity of the British Nurse?

**Published:** 1918-07-13

**Authors:** 


### THE REGISTRATION OF NURSES.

### By What Process Would Registration Contribute to the Prosperity of the British Nurse?

SCHOOL-CHILDREN, inspired with honest desire to understand, have a way of asking questions which superior people regard as foolish. I well remember inquiring why I should have to learn Latin grammar, and you can probably recall some similar experience. These tread-mill tasks appeared to have been designed to make child life miserable, and the worst of it was that nobody ever, or hardly ever, explained the secret. The child groping in the dark, and trying hard to take an intelligent view of life, is reprobated for asking foolish questions instead of meekly obeying orders. "Theirs not to reason why!"

IERNE IN A QUANDARY.

I am unfortunately in a quandary, and I seek information. But the same old feeling comes over me that came when I wore swaddling clothes. I am nervous about asking questions that are improper. I feel as if I am going to have my ears boxed, and be shut up in a dismal back room to discover in silence and solitude what everybody knows without being told. So nervous am I, and so nervous have I been, that I have spent toilsome hours in public libraries trying to discover secrets that everybody save myself seems to know. I wonder if anybody sympathises with this feeling. If your neighbour in the car or train remarks "We want more ships," and if you say "Why?" you will probably receive no answer. You are reasonably expected to know, and if everybody has to explain from the beginning of things conversation must automatically end. This is exactly where I am. The question that I have to ask is so elementary and so universally understood that I am sure nobody will reply, or even refer me to a primer where with the parts of speech and a definition of the imperative mood I might peradventure find what I seek.

WHAT MANY NURSES WANT TO KNOW.

I simply want to know what benefit State Registration will bring to nurses. I have blurted it out, and if the question is too ridiculous I humbly ask for pardon. Let me hasten to add that I am not opposed to State Registration. On the contrary, I regret extremely that it is not the law of the land, for then we should have done with it. I read the other day an account of a public meeting about nursing affairs, and when all the nurses' troubles had been gone over, Mrs. Bedford Fenwick, who was present, pointed out that they were all due to one outstanding trouble—the lack of State Registration. I read on filled with hope that at last the secret was about to be divulged; and indeed it may have been, but if so, the reporter or the editor cut it out. Then I see that M.P.s like Sir Arthur Stanley and Major Chapple discuss the subject with hope and fear, but not one of them ever tells. There is a conspiracy of silence, and so disappointment dogs my heels. I read about this Bill and that, which are on paper, and which I have carefully studied, and there is also the other Bill, not yet born, called "an agreed Bill." Lord Knutsford, I understand, abhors all Registration Bills for reasons unexplained.

REAL OR METAPHORICAL CHASTISEMENT INVITED.

Now, I do not in the least mind being abused. Write me down an ass by all means, or box my ears. Real or metaphorical chastisement I am willing to receive, but for pity's sake enlighten my darkness. I am honestly and earnestly anxious to join the procession, but I am afraid to do so lest somebody like myself should turn round and ask me awkward questions. Some hard-worked ward sister might give me a friendly nudge and ask—"Suppose it passes into law. What then?" If this happens I am stumped, for, honestly, I do not know. No Bill that I ever read or heard discussed proposes to reduce her hours, or increase her pay, or provide her with a pension, or in any respect to add a single cubit to her stature. No such Bill suggests the opening up of new fields of enterprise. All that it proposes to accomplish is to provide a list giving the names and addresses of women who have undergone a satisfactory period of training. I am not sure that the addresses would be given, but they may be.

IF WE HAVE AN AGREED BILL, WHAT THEN?

Let us suppose, for argument's sake, that to-morrow morning we have an agreed Bill, and that King, Lords, and Commons convert it into an Act, what then? So far as I can see the only result that would ensue is that we should have a little less noise, which of course would be something. Mrs. Bedford Fenwick says that we should then become what we are not now, a Profession. And here, again, I am thrown back on my native ignorance. What is a Profession? These are strenuous days of war, and people's brains become addled from too much thinking. I fear that I am in this unfortunate condition, but I have honestly striven to find light and leading. I suggested recently to a friend that I should like to see this elementary matter discussed in print. But, alas! she afforded me no consolation, but hinted that such an inquiry as I am taking the liberty of making would be "indiscreet." Nevertheless I am running the gauntlet, and have stiffened my back for the drubbing that I presume I must receive.

KATHLEEN MAVOURNEEN'S FATE FOR REGISTRATION?

If legislation were impending I should suppress my curiosity and wait to see what would happen. But I foresee that it may be long in coming. A kind of Kathleen Mavourneen fate seems to hang over all these Bills, and a pessimist might be forgiven for prophesying that King, Lords, and Commons, as we know them, may pass hence without satisfying our ambitions. Are we in such circumstances to continue shouting, or had we better seek for ourselves dishonourable graves? Meanwhile perhaps somebody more thoughtful and considerate than the rest will for the benefit of people like myself, who are only stupidly but not invincibly ignorant, be so kind as to explain in a few simple sentences how and by exactly what process State Registration, if and when it does come to pass, will contribute to the prosperity of the British nurse.

